# Sex Differences in Neurophysiological Changes Following Voluntary Exercise in Adolescent Rats

**DOI:** 10.3389/fneur.2021.685822

**Published:** 2021-07-22

**Authors:** Lindsay Ferguson, Christopher C. Giza, Rebecka O. Serpa, Tiffany Greco, Hannah Robert, Michael Folkerts, Mayumi L. Prins

**Affiliations:** ^1^Department of Neurosurgery, Brain Injury Research Center, David Geffen School of Medicine, University of California, Los Angeles, Los Angeles, CA, United States; ^2^Steve Tisch BrainSPORT Program, University of California, Los Angeles, Los Angeles, CA, United States; ^3^Department of Psychology, Seaver College, Pepperdine University, Malibu, CA, United States

**Keywords:** adolescence, exercise, sex differences, BDNF, estrous cycle

## Abstract

**Background:** Adolescence is a period of time characterized by the onset of puberty and is marked by cognitive and social developments and gross physical changes that can play a role in athletic performance. Sex differences are present with differences in body size, height, physiology and behavior which contribute to differences in athletic performance as well. Pre-clinical studies representing this active group are lacking.

**Methods:** Acute and chronic effects of exercise were evaluated. Male and female adolescent rats were given voluntary access to a running wheel for 10 consecutive days. Running behavior (males and females) and estrous cycling (females only) were analyzed daily. A second group was given 10 days of voluntary access to a running wheel, then rested for 10 days to determine the long-term effects of exercise on the adolescent brain. Brain and muscle tissue were harvested at 10 and 20 day time points to understand exercise-dependent changes in mitochondrial activity and neuroplasticity. Animal cohorts were carried out at two different sites: University of California Los Angeles and Pepperdine University.

**Results:** On average, running distance, intensity of run, and length of running bout increased for both male and female rats across the 10 days measured. Females ran significantly further and for longer intervals compared to males. Cortical and muscle expression of PGC1α showed similar levels at 10 days regardless of sex and exercise. There was a significant increase in expression at 20 days in all groups correlating with body size (*p*'s < 0.05). Cortical and hippocampal levels of BDNF were similar across all groups, however, BDNF was significantly higher in exercised females at the acute compared to long-term time point.

**Discussion:** Adolescent rats allowed 10 days of exercise show changes in physiologic function. There are sex differences in running behavior not impacted by sex hormones. These results are important to further our understanding of how exercise impacts the adolescent brain.

## Introduction

Adolescence is a period of time characterized by the onset of puberty and the acquisition of social behaviors necessary for survival and reproduction (1). Adolescence is also marked by gross physical changes that can play a role in athletic performance in addition to cognitive and social developments. Maturational changes and physical activity can influence one another in a bidirectional manner. Puberty includes sexual maturation, secretion of sex hormones, and reproductive function. Puberty is a sexually dimorphic process that begins earlier in biological females. This earlier onset can result in differences in body size, height, physiology and behavior which contribute to differences in athletic performance (2). Specifically, growth and behavioral changes that occur during this time are not in tandem and can independently effect both an adolescent's athletic trajectory and competitive performance (3). In rodents, body size and gonadarche has been linked to pubertal onset as well (4–6). However, while puberty in humans and chimpanzees is characterized by adrenarche, the same does not occur in rodents (7). Understanding the differences between rodent and human development, particularly during adolescence, is important for translation.

In the larger context of neurotrauma, understanding exercise-dependent changes on the adolescent brain can help to uncover the recovery profile of adolescents following brain injury. Despite adolescent athletes being at high risk for mild traumatic brain injury (TBI), the majority of pre-clinical mild TBI studies have not considered exercise pre-conditioning as a variable (8, 9). Further, studies suggest that female athletes suffer concussion at higher rates compared to males in similar sports and recovery from TBI may have some sex-dependence (10). Physical exercise reduces the risk of cardiovascular disease, stroke, and hypertension, and also results in changes of metabolic function, stress response, and neuroplasticity. These mechanisms are also interrupted following TBI emphasizing the need to understand the baseline functional differences of athletes.

Athletic performance in females may also contribute to sex hormone dysfunction. Distance running has been associated with irregular menstrual cycling and delayed menarche and pubertal onset in adolescent females, and increased distance run has been correlated with increased menstrual cycle irregularity (11, 12). Female athletes are particularly at risk for low energy availability potentially due to disordered eating, low bone mineral density, and menstrual dysfunction, commonly known as the Female Athlete Triad (13, 14). In particular, sports that promote a lean body type (gymnastics, cheerleading, etc.), tend to increase a female's risk for one or more components of the Triad (14). The concept of the Female Athlete Triad has been expanded to include males and non-athletes, and is now known as Relative Energy Deficiency in Sports (RED-S) (15, 16). The important inclusion of males emphasizes the significance of energy availability in normal physiological function. Low energy availability due to excessive exercise and low caloric intake diverts energy expenditure away from processes not necessary for immediate survival, like growth, fat accumulation, and development (16). These changes are necessary to take into consideration when studying the adolescent athlete, particularly in females. In rodent studies, measuring growth is an important component as it may indicate low energy availability and decreased fat accumulation. Also, monitoring estrous cycling is an important aspect to include in females given the Female Athlete Triad.

Exercise has many benefits. It has been shown to release peroxisome proliferator-activated receptor gamma coactivator 1-alpha (PGC1α) and brain-derived neurotrophic factor (BDNF) in skeletal muscle and leads to the release of both in the brain, particularly the hippocampus, in rodent and human studies (17, 18). BDNF expression facilitates neurogenesis, neuroprotection and cognitive enhancement. PGC1α seems to be imperative for BDNF expression as PGC1α knock-out mice do not show increases in BDNF in the brain (17). The acute and chronic changes in BDNF concentrations that influence brain function seem to differ based on sex, age and training experience. These factors are important to understand at baseline as it may influence outcomes following TBI and other traumas.

Typically, exercise regimens are performed over a course of weeks or months. In adult humans, 3 months of endurance training raised resting state plasma BDNF but remained stable following 30 min spurts of exercise (19). Physical exercise over 6 weeks in young adult males found increased serum BDNF that positively correlated with hippocampal volume and post-exercise serum BDNF (20). In young adult mice, 5 weeks of treadmill running increased BDNF mRNA in the hippocampus alone (19). Pre-clinical studies focused on adolescence are limited in time as the adolescent period is only about 30 days in rats. As such, long periods of time for exercise training are not possible. This is particularly true if studies are interested in evaluating recovery from TBI. As exercise has been shown to increase cognition and memory and BDNF expression, it's important to understand how much exercise is necessary to observe beneficial changes in the brain. One study on adults found that 6 days of voluntary running was sufficient to see increases in BDNF in the hippocampus (21). Another experiment evaluating 21 days of voluntary running wheel exercise in both male and female adolescent mice found increased BDNF mRNA expression in the hippocampus (specifically CA1 region) in both sexes (22). More research in this area is necessary to understand the effects of exercise on adolescents and differences that could occur as a result of sex, such as following TBI. The current study assessed a 10 day voluntary running paradigm and analyzed acute (10 day) and chronic (20 day) changes in physiology.

In the current study, adolescent male and female rats were given voluntary access to a running wheel. Sex was an essential component with analysis during various stages in the female estrous cycle. Exercise activity, body weight, and menarche onset and pattern was recorded for each individual rat. This study aimed to determine if 10 days of exercise was sufficient to observe changes in PGC1α and BDNF in brain and skeletal muscle in adolescents. A second aim was to determine effects of exercise on cycling patterns in females. It was hypothesized that 10 days of voluntary running wheel exercise is sufficient to see enduring physiological changes in exercised adolescent rats.

## Materials and Methods

In response to the National Institute of Health's initiative to enhance reproducibility across laboratories, behavioral collection of animals was carried out at two different sites: University of California Los Angeles (Los Angeles, CA) and Pepperdine University (Malibu, CA). Data from these sites were expected to increase heterogeneity in the rat population. Characteristics of the sites that have shown to contribute to differences in animal population and results are described below (23, 24).

### Pepperdine

Housing and experimental environments were used solely by one laboratory. Humidity ranged from 35–78%. Male and female rats arrived at the facility at 28 days of age and acclimated to their environment for 7 days before daily handling. Experimenters were the only ones to handle rats throughout the study. Rats of same sex were housed in groups of 3 on a 12:12 light: dark cycle with lights on at 06:00. Standard chow (Lab Diet 1,5001, Newcolab, CA) and water was available *ad libitum*.

### University of California Los Angeles

Housing and experimental environments were shared by four experimenters. Humidity was within the 30–70% range. Male and female rats arrived at the facility at 28 days of age and acclimated in a quarantine room with rats from a variety of sources. At 31 days of age they were transferred to a different and permanent housing room and handled daily by both experimenters and animal care staff. Rats of the same sex were housed in groups of 4 on a 12:12 light: dark cycle with lights on at 06.00. Standard chow (Teklad, Enivigo, WI) and water was available *ad libitum*.

### Subjects

Eighty-eight adolescent male (110–170 g) and female (110–140 g) Sprague Dawley rats were 35 days old at the start of study at both research sites (Charles River, RRID:SCR_003792). Animals were housed in sex specific groups of 3 or 4 throughout the entire study on a 12:12 light: dark cycle with lights on at 06:00. Food and water was available *ad libitum*. Females were monitored daily between 11:00–13:00 to determine stage in estrous cycle. Vaginal opening occurs in female rats between post-natal days (PND) 34–37, and therefore not all animals had an active estrous cycle on day 1 of the study (*n* = 12). It is important to note that there is no equivalent procedure for males. While vaginal lavage is an accepted procedure for females, the daily handling may be a confounding variable in stress and corticosterone assays. All procedures were approved by The UCLA Chancellor's Animal Research Committee and National Institute of Health Guide for the Care and Use of Laboratory Animals and the Institutional Animal Care and Use Committee of Pepperdine University.

**Figure 1:**
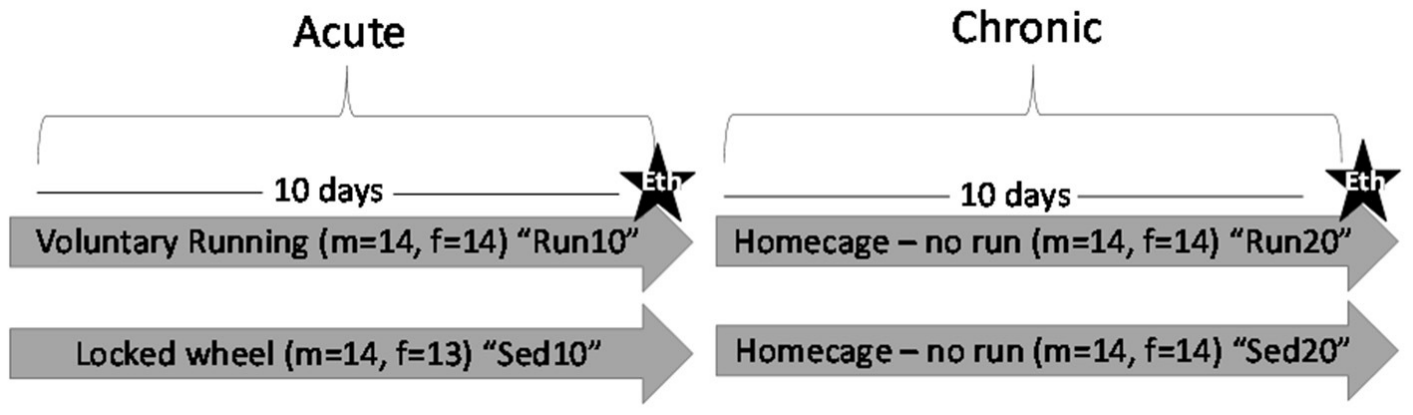
Experiment Outline. Male (m) and female (f) rats were placed in a locked or functional running wheel for 10 days and then euthanized (star) to determine acute effects of exercise. A 2nd cohort of rats were placed in a functional or locked running wheel for 10 days then rested in their homecage for an additional 10 days before euthanasia (star) to determine chronic changes due to exercise. Eth, euthanasia.

Beginning on PND35, males and females were placed into individual cages containing a locked (sedentary) or functional (rathlete) running wheel (Starr Life Sciences, Oakmont, PA) from 22:00–06:00 the following day. Running wheels were located in same room as housing and light:dark cycle was same as previously mentioned. Food and water were available during this time. Each morning, animals were placed back in their homecages with the same social groups. The number of revolutions was recorded each minute over this time using VitalView v5.1 (VitalView Software, RRID:SCR_014497). Average distance (m), intensity (m/min), and intervals (min/bout) of running were determine for each animal on each day. One group of males and females were euthanized after wheel removal on the 10th day. A 2nd group was monitored for an additional 10 days (socially housed, no running wheels) to determine the stability of neurochemical changes (Figure 1). Animals were randomly assigned to the four total groups.

### Estrous Cycle Determination

Each day estrous cycling in females was monitored by vaginal lavage using sterile normal isotonic 0.9% saline as has been previously described (25). Briefly, 0.2 ml of sterile saline was used to flush the vaginal opening. When inserting the syringe tip into the opening, care was taken to not insert the syringe further than the tip (2 mm) to prevent cervical stimulation and pseudopregnancy. Samples obtained were then characterized microscopically by appearance of cell types (leukocytes, nucleated or round epithelial cells, or keratinized) and cycle was described as (1) proestrus (small nucleated epithelial cells, arranged into clusters, sheets, or strands, and non-viscous and transparent appearing vaginal fluid), (2) estrus (keratinized, anucleated cells, and non-viscous and transparent vaginal fluid), (3) metestrus (leukocytes and round epithelial cells, vaginal fluid remains non-viscous and transparent), or (4) diestrus (majority leukocytes, vaginal fluid can be observed as viscous and opaque). Typically, rodent cycles are 4–5 days in length, with estrus lasting 1 day (4 day cycle) or 2 days (5 day cycle). Proestrus typically lasts 12 h, estrus last 24–48 h metestrus lasts 6–8 h, and diestrus makes up the remaining time (2–3 days). Cycling was observed for regularity. “Regular” was defined as a 4 or 5 day cycle with 1–2 days of estrus followed by 2–3 days of metestrus and diestrus. Irregular cycles were classified as either “extended” or “abnormal.” “Extended” was defined as 3–4 consecutive days of estrus or 4–5 consecutive days of metestrus/diestrus. “Abnormal” was defined as cycles <3 days, more than 4 consecutive days of estrus, or more than 6 consecutive days of diestrus. Cycle length was determined following vaginal opening from the 1st day of diestrus through the last consecutive day in estrus. Average days in estrus per cycle was also determined. As the cycles of each individual subject may not align with the typical 4-day model or categorization determinants, the ability to recognize what is seen as typical for each subject is important. *Therefore, rather than comparing samples from different subjects, it becomes necessary to solely compare samples from the same subject when attempting to categorize, as individual differences within stages occur and can lead to misidentification*. To decrease the amount of bias and subjectivity involved in this process, two researchers conferred in the categorizing process.

### End Point

Subjects were euthanized between 09:00 and 12:00 on day 11 to determine acute effects of exercise on physiological measures, or on day 21 to determine chronic effects. Subjects were lightly sedated using 5% isoflurane and 2% oxygen followed by rapid decapitation using a guillotine. Trunk blood was collected using ethylenediaminetetraacetic acid coated capillary tubes (Instech Labs, Plymouth Meeting, PA) and spun at 4°C and 15,000 rpm for 20 min. Plasma supernatant was collected and stored at −80°C for analysis. The brain was rapidly removed over ice. One hemisphere was isolated into hippocampal and cortical sections and stored at −80°C for Western blot analysis. The gastrocnemius muscle was also removed and stored at −80°C for Western blot analysis.

### Western Blot

Brain and muscle tissue were homogenized in radioimmunoprecipitation assay (RIPA) buffer (for BDNF, Thermo-Fisher, Waltham, MA) or nuclear extraction buffer (NE-PER, for PGC1α, Thermo-Fisher) to denature proteins. They were spun for 20 min at 30,000 g at 4°C and the supernatant was removed. Total protein concentration was determined using Bio-Rad BCA protein assay. Following, 20 mg was loaded into each well of a 17 well-acrylamide gel (4–12%, Thermo-Fisher). Protein bands were separated at 200 v for 50 min. Following they were transferred to a polyvinylidene difluoride (PVDF) membrane. SYPRO Ruby Protein Blot Stain (Thermo-Fisher) was first run to visualize the efficiency of the protein transfer and to normalize target band against total protein (Supplementary Figure 1). Membranes were blocked in 5% milk for 1 h, washed in tris-buffered saline (TBS), then incubated in primary antibody against brain derived neurotrophic factor (BDNF, 1:2000, Novus, Centennial, CO, USA, Cat# NB100-98682, RRID:AB_1290643) and anti-peroxisome proliferator-activated receptor gamma coactivator 1-α (PGC1α, 1:5000, Abcam, Cambridge, MA Cat# ab54481, RRID:AB_881987) overnight at 20°C. Samples were then washed in TBS+ 0.1% Tween 10 and incubated for 1 h in secondary anti-rabbit IgG (1:20000 Vector Laboratories Cat# BA-1000, RRID:AB_2313606) at room temperature. Bands were visualized using ECLplus kits (Thermo-Fisher). BDNF band was read at 28kDa and PGC1α was read at 102kDa.

### BDNF Antibodies

BDNF results in multiple bands when separated into its cleavage products. Analysis of specific bands may provide different results and conclusions. To test this, samples from rathlete and sedentary groups were analyzed with three different BDNF antibodies, from three different companies (Novus Cat# NB100-98682, RRID:AB_1290643, Abcam Cat# ab108319, RRID:AB_10862052, Santa Cruz Biotechnology Cat# sc-546, RRID:AB_630940), targeting three different bands in a Western blot (Supplementary Table 1). Included in this is the Novus BDNF antibody used in the main analysis and described in the previous section.

### Corticosterone

As this is a test of stress hormones, timing of euthanasia and animal handling procedures can influence results. There were slight differences between sites. At UCLA, rats were moved from a housing room to a holding room on a different floor. Euthanasia occured in a room separate from the holding room as well, with a wooden door separating rooms to minimize odors. Rats were carried to the euthanasia room individually just prior to euthanasia. At Pepperdine, housing room was in same location as euthanasia. Rats were individually taken from their home cage into a separate room for euthanasia. Rats from each group were euthanized throughout the 3 h process making variability in timing consistent across groups. Rats were lightly sedated for 2–3 min before rapid decapitation. Plasma samples were analyzed for corticosterone using a 96-well-plate coated with donkey anti-sheep IgG ELISA kit (lot: 19A663, Invitrogen, Carlsbad, CA). Inter-assay variability ranged from 6.6–7.5%CV. Samples were prepared with 5 μL dissociation reagent and diluted 1:100 using Assay Buffer. All samples were analyzed simultaneously. Each well was loaded with 50 μL of each sample and standards ranging from 10,000–0 pg/mL. Then, 25 μL of corticosterone conjugate was added to each well, followed by 25 μL of corticosterone antibody. One well of each plate was loaded with 75 μL Assay Buffer to detect non-specific binding and 25 μL corticosterone conjugate. Next, 100 μL of tetramethylbenzidine solution was added and samples incubated for 30 min. Finally, 50 μL of Stop solution was added to each well. Absorbance was read immediately at 450 nm. Sample concentrations were determined from the standard curve.

### Statistics

Analysis of the rathlete model was evaluated using GraphPad Prism v 9.0.2 (GraphPad Prism, RRID:SCR_002798) and R v 3.6.0 (R Project for Statistical Computing, RRID:SCR_001905). Body weight was compared between rathlete and sedentary groups separately for males and females using a Student's *t*-test. Male and female rats were analyzed for running behavior (distance, intensity, interval) using mixed-effects ANOVA with day as the repeated measure, followed by Sidak-corrected *post-hoc* comparisons. A linear regression was performed to compare changes in running behavior over time separately for males and females, and slopes were compared. Weight gain, expression of BDNF and PGC1α protein levels were compared at the acute 10 day and chronic 20 day timepoint using a Two-way ANOVA for activity (running vs. sedentary) and sex (male vs. female). Correlations between body weight and PGC1α were also compared using Pearson's correlation coefficient (normally distributed data) or Spearman's test. Estrous cycling pattern categorizations (regular, irregular) in females were compared separately for sedentary and rathlete groups using a Fisher's exact test to determine effect of activity on cycling regularity. Length of cycles and days in estrus were compared using aStudent's *t*-tests or Mann-Whitney *U*-tests, if test of normalcy failed. Estrous stage was also compared to running behaviors using Spearman correlation, as data was not found to have a normal distribution.

## Results

### Site Reproducibility

Data were pooled between sites. Animals were housed and handled at Pepperdine and UCLA, and have different laboratory characteristics which have previously been shown to add variability to groups (24). Males from both sites showed similar daily weight, weight gain, distance run, run intensity, and running intervals. Females, too, showed similar daily weight, weight gain, average run intensity, and interval run across the 10 days measured. However, females from Pepperdine showed greater daily increases in running distance (bigger linear regression slope) than that observed in females at UCLA.

### Males

Body weight increased steadily from 136.8 ± 3.7 g to 210.2 ± 4.3 g over 10 days of running and to 270.8 ± 7.1 g over 20 days. Weight of sedentary rats increased from 136.4 ± 3.4 g to 217.1 ± 2.9 g over 10 days and to 287.7 ± 4.8 g over 20 days. No significant differences were found between sedentary and rathlete groups [*t*_(38)_ = 0.809, *p* = 0.423].

### Male Rathlete

**Figure 2:**
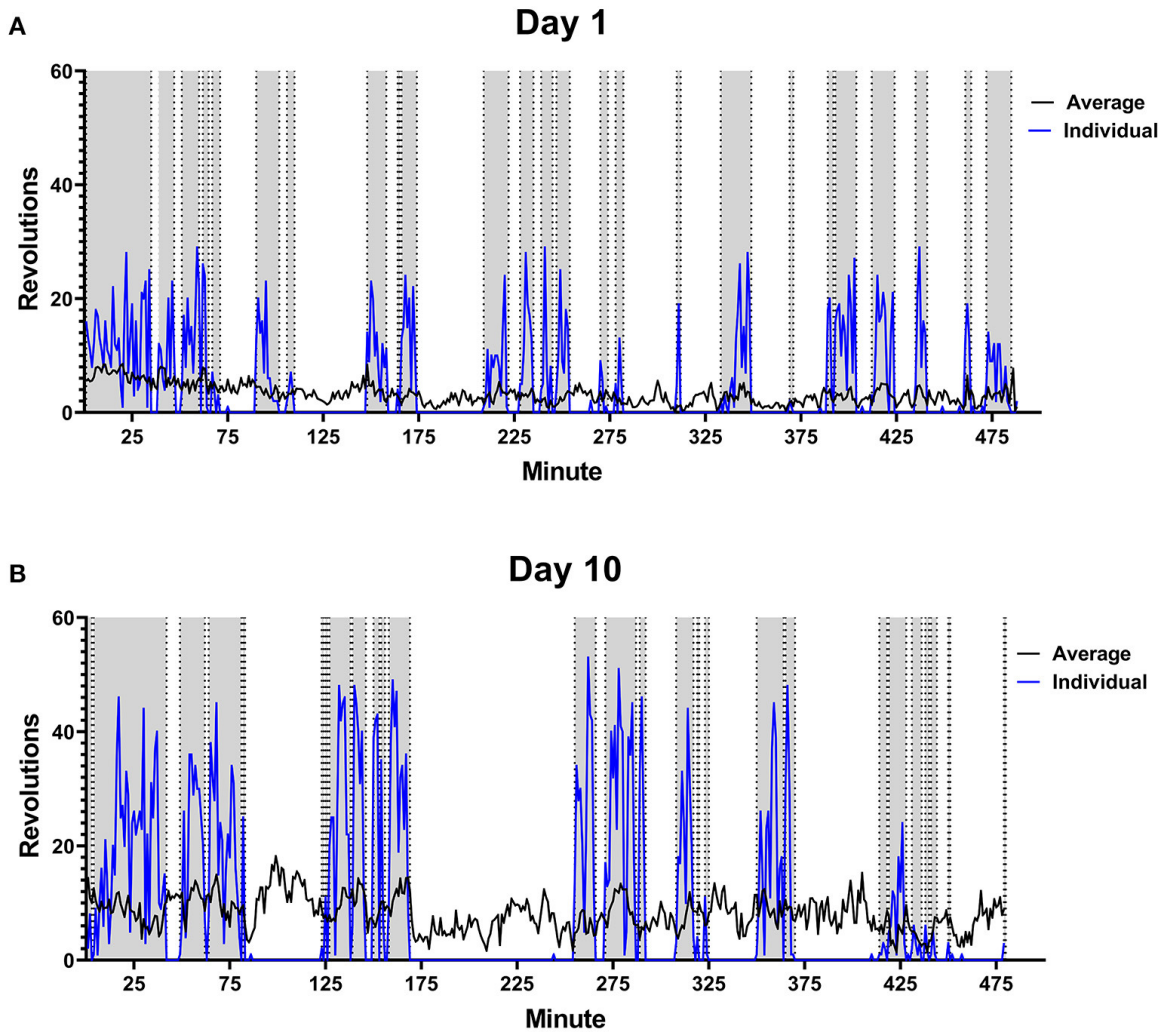
Male Rat Running Pattern. Rats were placed in a cage with voluntary access to a running wheel from 10:00–06:00 (480 min) for 10 days. An example is shown of the running pattern of one male in the study on Day 1 **(A)** and Day 10 **(B)** of wheel access. Blue line is the number of revolutions run by the individual rat. The gray portions highlight running intervals. Black line indicates average distance run by all male rats on each perspective day (*n* = 28).

Running behavior was analyzed over the 8 h time period for each rat daily. Total distance run, average intensity of running period, and average length of running per bout was calculated each day for each subject. Rats given voluntary access to functional running wheels ran each day of testing. Distance run each day increased consistently from 1648 ± 182.1 m to 4363 ± 595.5 m, as did intensity of running from 7.69 ± 0.5 m/min to 20.71 ± 1.38 m/min. Length of running bout remained consistent over 10 days. The pattern of running from one rat is provided in Figure 2 to illustrate changes between day 1 and day 10.

### Female

Rathlete weights increased from 124.0 ± 2.1 g to 175.3 ± 2.8 g over 10 days and to 203.9 ± 6.5 g at 20 days. Sedentary rats weighed 123.7 ± 2.4 g at the start, and increased to 168.3 ± 3.1 g at 10 days and to 200.2 ± 4.5 g at 20 days. No significant differences were found between rathletes and sedentary groups [*t*_(38)_ = 0.410, *p* = 0.684].

### Female Rathlete

**Figure 3:**
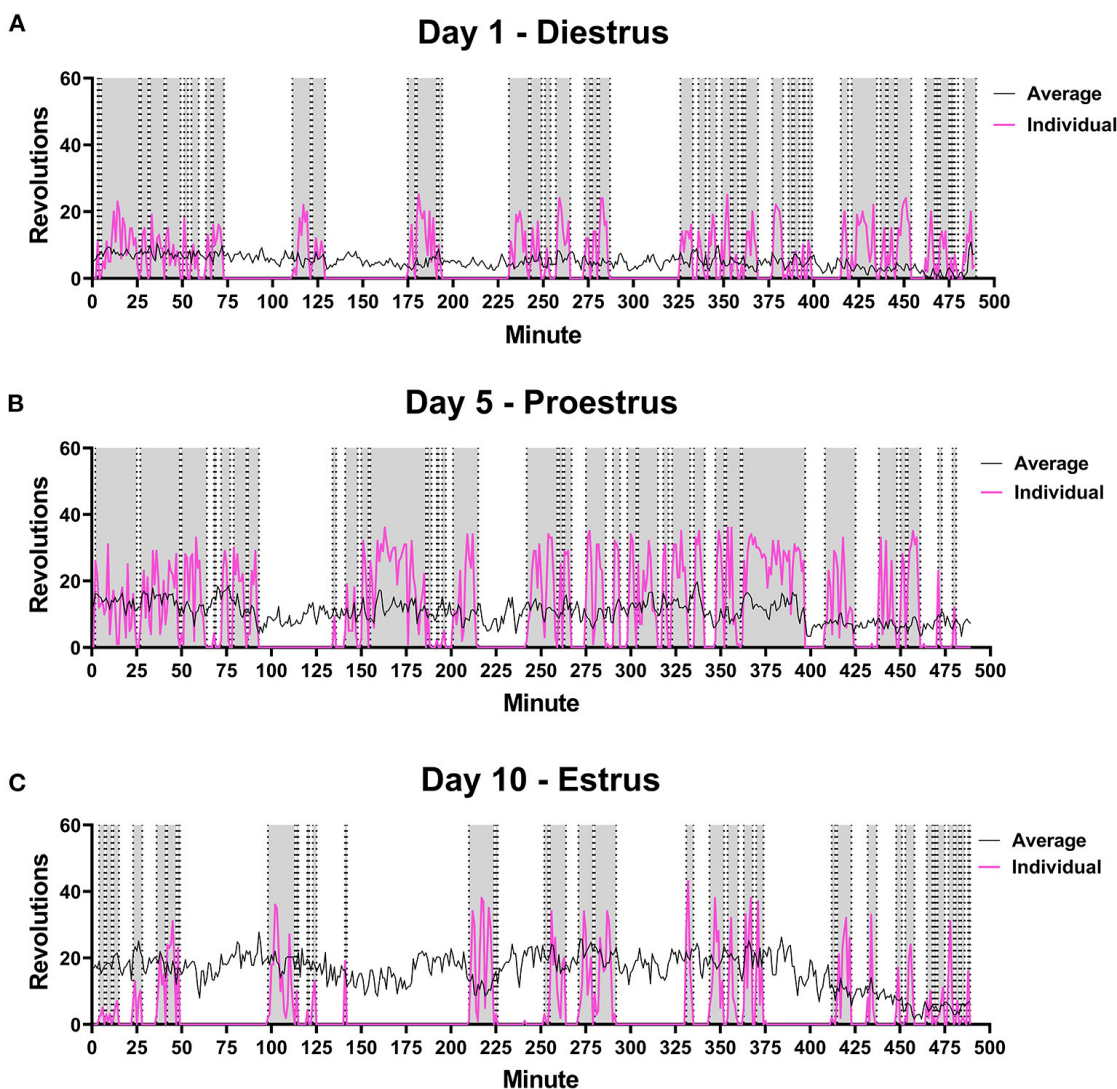
Female Rat Running Pattern. Rats were placed in a cage with voluntary access to a running wheel from 10:00–06:00 (480 min) for 10 days. Stage in estrous cycling (Diestrus, Proestrus, Estrus) was monitored upon removal from wheels. An example is shown of the running pattern of one female in the study on Day 1 **(A)**, Day 5 **(B)** and Day 10 **(C)** of wheel access. Distance run was compared to estrous stage, but no correlations were observed. Pink line is the number of revolutions run by the individual rat. The gray portions highlight running intervals. Black line indicates average distance run by all female rats on each perspective day (*n* = 28).

**Figure 4:**
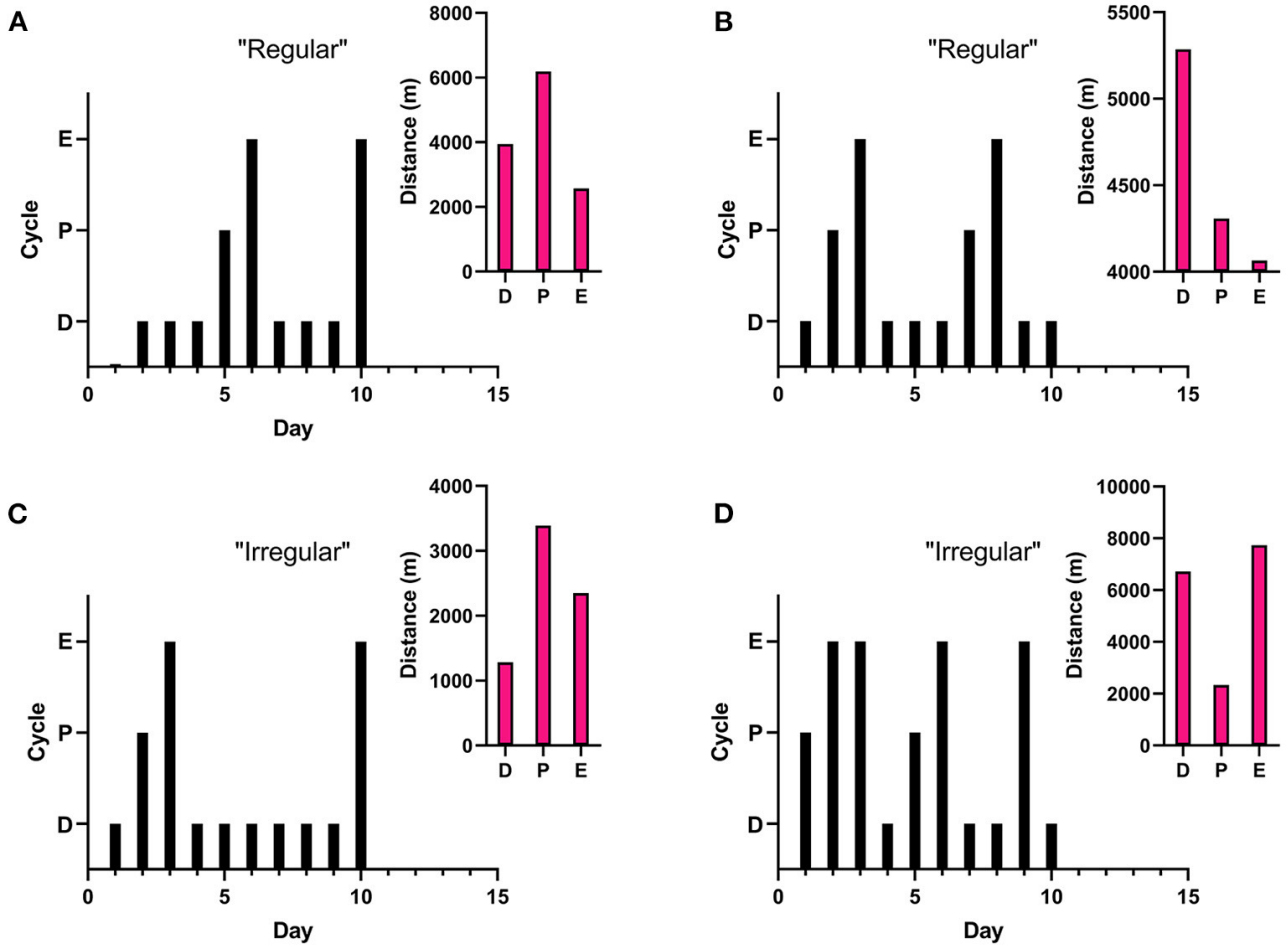
Estrous Related Running. Stage in estrous cycling (Proestrus, Estrus, Diestrus) was monitored each day rats were in locked or functional wheels. A “regular” cycle is a typical 4–5 day cycle (estrus to estrus), while an “irregular” cycle can be either extended (3–4 consecutive days in estrus or 4–5 consecutive days in diestrus) or abnormal (cycle length <3 days, >4 days in estrus, or >6 days in diestrus). Graphs represent cycling patterns of individual female rats. Black bar graphs represent cycling patterns of individual females with the lowest bars representing diestrus (“D”), the middle bars representing proestrus (“P”), and the tallest bars representing estrus (“E”). The pink insets show representative distance run of the same rat during different cycling stages. **(A)** Regular cycle with running distance correlating with estrous stage. **(B)** Regular cycle without correlation with estrous stage. **(C)** Irregular (extended) cycle with running distance correlating with estrous stage. **(D)** Irregular (abnormal) cycle without correlation with estrous stage.

On average, running distance, intensity of run, and length of running bout increased across the 10 days measured. Individual running patterns fluctuated over these days for each rat, as opposed to consistently increasing in males. An example from one rat is provided in Figure 3 in regards to estrous cycle stage of rat and day of run. For some individual rats, the distance run was related to stage of estrous cycle. This was not true of the overall average, however (see Figure 4). There was no significant correlation between stage in estrous to distance [*r* = 0.08, *p* = 0.23], intensity [*r* = 0.09, *p* = 0.17], nor length of running interval [*r* = 0.09, *p* = 0.22]. The lack of correlation was not due to regularity in cycling. Both regular and irregular cycling subjects were as likely to run further distances during proestrus as they were likely to not.

### Estrous Cycling

Estrous cycles were monitored for each subject and patterns were analyzed. Vaginal opening had not yet occurred on the 1st day of monitoring in 5/22 (23%) rathletes. For sedentary females, 7/21 (33%) did not have observed vaginal opening on the 1st day of estrous cycle monitoring. A chi-square analysis was performed to compare the number of cycles classified as regular, extended, or abnormal. No differences were seen between sedentary and rathletes [two-sided fisher's test, *p* = 0.25] indicating that the rathlete model is not affecting estrous cycling. There were also no differences between groups for days in estrus over the 10 days measured [*t*_(41)_ = 0.28, *p* = 0.778] nor cycle length [Mann-Whitney *U*-test = 210.5, *p* = 0.81].

### Sex Differences

**Figure 5:**
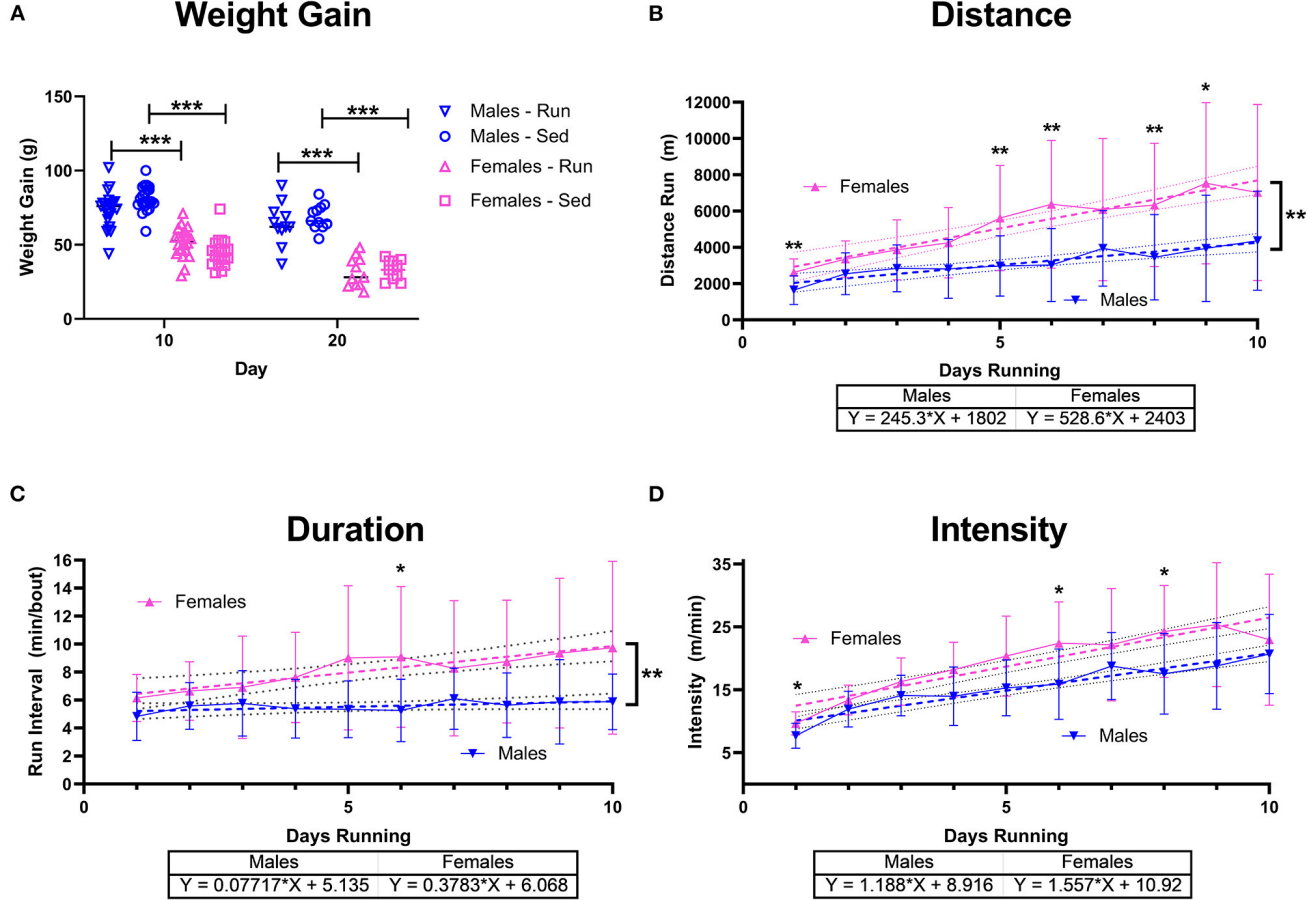
Sex Differences with Running. Male (blue) and female (pink) rats were placed in a locked or functional running wheel for 10 days to determine acute effects of exercise (*n* = 14/sex). A 2nd cohort of rats were placed in a functional or locked running wheel for 10 days then rested in their homecage for an additional 10 days (*n* = 14/sex). **(A)** Weight gain at the 10 day and 20 day time points were measured. **(B)** Distance run was calculated daily and across 10 days of running. **(C)** Duration of running periods and **(D)** intensity of running was also calculated daily. Data are presented as average ± SD. A linear regression (dotted lines) was performed on each to determine change across time and compared between males and females and equation of line is provided. *p < 0.05, **p < 0.01, ***p < 0.001.

After subjects from UCLA and Pepperdine were combined, sex differences were analyzed. Weight gain showed an activity x sex interaction [*F*_(3,123)_ = 119.7, *p* < 0.001]. Sedentary and rathlete females gained significantly less weight than males at both the acute and chronic time point [Sidak-corrected *post hoc t's*_(123)_ = 3.47–11.23 *p*'s = 0.001–0.02] (Figure 5A).

Running behavior also revealed sex differences. A significant time x sex effect on distance run was observed [Mixed-effects ANOVA: *F*_(9,346)_ = 3.20, *p* = 0.001] with females showing greater distance run on days 1 [Sidak-corrected *pots-hoc*: *t*_(37)_ = 4.00, *p* = 0.003], 5 [*t*_(32)_ = 3.60, *p* = 0.01], 6 [*t*_(32)_ = 3.79, *p* = 0.006], 8 [*t*_(36)_ = 3.16, *p* = 0.03], and day 9 [*t*_(33)_ = 2.23, *p* = 0.03] (Figure 5B). A linear regression also indicated a significant increase in daily distance run for both males [*F*_(1,196)_ = 25.58, *p* < 0.001] and females [*F*_(1,208)_ = 49.66, *p* < 0.001], though females increased to a greater extent than males [F_(1, 404)_ = 9.78, *p* = 0.002]. Similarly, running intervals indicated a significant time x sex effect [*F*_(9,346)_ = 2.24, *p* = 0.02] with females running for longer intervals on day 6 [*t*_(28)_ = 3.18, *p* = 0.04] (Figure 5C). A linear regression also indicated significant sex differences [*F*_(1,405)_ = 6.43, *p* = 0.01] with females increasingly running for longer bouts compared to males. Intervals of running only increased in females [Mixed –effects ANOVA: *F*_(1,208)_ = 13.40, *p* < 0.001] not males [*F*_(1,296)_ = 2.09, *p* = 0.15]. Intensity of run also indicated a significant effect for sex [*F*_(9,346)_ = 2.23, *p* = 0.02] on days 1 (Sidak-corrected *post-hoc*: *t*_(37)_ = 3.23, *p* = 0.02], day 6 [*t*_(39)_ = 3.46, *p* = 0.01], and day 8 [t_(39)_ = 3.29, *p* = 0.03], though linear regression did not show differences [*F*_(1,404)_ = 3.06, *p* = 0.08] between groups indicating a similar overall increase in intensity over 10 days of voluntary running [males: *F*_(1,196)_ = 90.09, *p* < 0.001, females: *F*_(1,208)_ = 87.42, *p* < 0.001] (Figure 5D).

### Protein Expression

**Figure 6:**
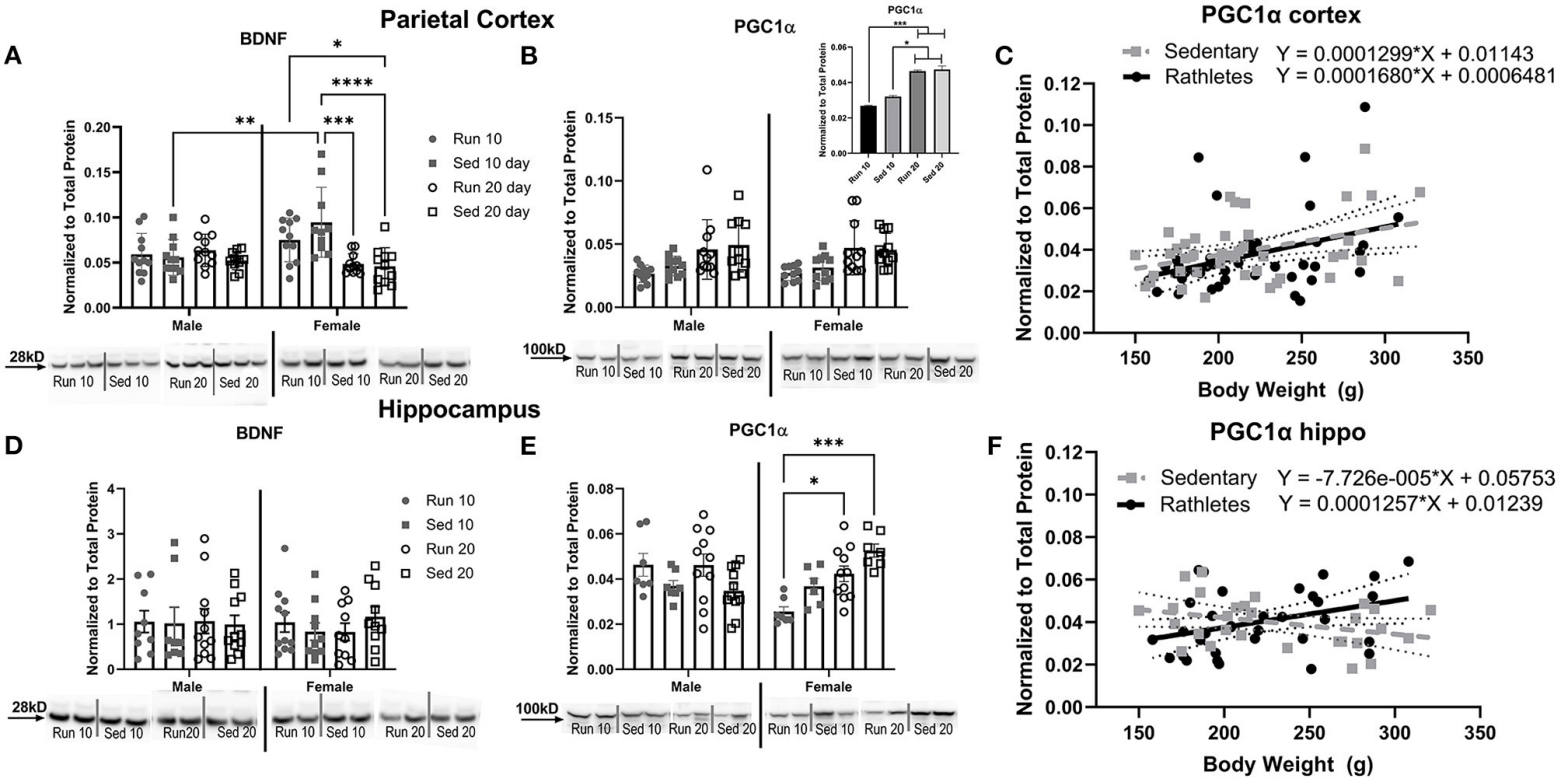
Protein Expression in Brain. A Western blot was used to determine levels of **(A)** BDNF and **(B)** PGC1α in the parietal cortex. Acute (10 day) and chronic (20 day) changes in protein expression were compared between rathletes (“Run”) and sedentary (“Sed”) rats between these time points. Sex differences were also analyzed. Where sex differences were not present, males and females were combined (**B**, inset). A correlation and linear regression were analyzed between body weight and density of PGC1α in the **(C)** cortex. Density of specific bands were normalized to total protein by Sypro Ruby staining (see Supplementary Figure 1). Hippocampal expression of BDNF **(D)** and PGC1α **(E)** was also determined, as well as correlations in body weight to PGC1α **(F)**. For correlations, males and females were combined within the rathlete and sedentary groups. Significant regressions were found for rathletes in the cortex (*p* = 0.02) and hippocampus (*p* = 0.04). Significance was only found for the sedentary group in the cortex (*p* = 0.02), but not hippocampus (*p* = 0.08). Run 10 = voluntary running wheels for 10 days (*n* = 14/sex), Sed 10 = locked wheels for 10 days (*n* = 14/sex), Run 20 = voluntary running for 10 days then rested in homecage 10 days (*n* = 14/sex), Sed 20=locked wheels for 10 days then rested in homecage for 10 days (*n* = 14/sex). Data are presented as average ± SEM. *p < 0.05, **p < 0.01, ***p < 0.001, ****p < 0.0001.

Acute (10 days) and chronic (20 days) effects of running on protein expression was determined. Rats were euthanized and brain tissue was analyzed in males and females for BDNF and PGC1α expression. In the parietal cortex, there were significant differences in BDNF relative to activity [Two-way ANOVA: *F*_(3,79)_ = 5.967, *p* = 0.001] and interaction with sex [*F*_(3,79)_ = 6.495, *p* < 0.001], but not for sex alone [*F*_(1,79)_ = 3.164, *p* = 0.79] (Figure 6A). Sedentary male rats at 10 days showed significantly less BDNF expression compared to sedentary females at 10 days [Sidak-corrected *post-hoc*: *t*_(79)_ = 4.05, *p* = 0.003]. No other significant differences between males and females were found. In females, sedentary rats at 20 days showed significantly less expression compared to female rathletes [*t*_(79)_ = 4.41, *p* = 0.05] and sedentary females at 10 days [*t*_(79)_ = 7.18, *p* < 0.001]. For PGC1α there were only significant differences for activity [*F*_(3,76)_ = 9.212, *p* < 0.001], but not for sex [*F*_(3,76)_ = 0.119, *p* = 0.95], or interaction [*F*_(1,76)_ = 0.062, *p* = 0.80] (Figure 6B). *Post-hoc* analyses for the main effect of activity found significantly higher expression of PGC1α at 20 days for rathlete and sedentary rats compared to rathlete [*t's*_(76)_ = 4.09–4.24, *p's* < 0.001] and sedentary rats [*t's*_(76)_ = 3.04–3.21, *p's* = 0.012–0.019] at 10 days (Figure 6B, inset). As exercise and sex did not appear to influence expression of PGC1α, a Pearson's correlation coefficient was calculated to determine relation of protein expression of PGC1α to body weight (Figure 6C). Rathlete and sedentary groups were combined separately across sex and time. A significant correlation was found in the cortex for both rathletes [*r* = 0.36, *p* = 0.02] and sedentary rats [*r* = 0.38, *p* = 0.01].

In the hippocampus, there were no significant differences in BDNF expression [Sex: *F*_(1,72)_ = 0.15, *p* = 0.70; Activity: *F*_(3,72)_ = 0.20, *p* = 0.89; Interaction *F*_(3,72)_ = 0.31, *p* = 0.81] (Figure 6D). There were, however, significant differences in PGC1α levels (Figure 6E). A Two-way ANOVA found a significant interaction with sex and activity [*F*_(3,59)_ = 7.91, *p* < 001]. In females, protein expression in rathletes at 10 days was significantly less than rathletes [Sidak-corrected *post-hoc*: *t*_(59)_ = 3.12, *p* = 0.01] and sedentary rats [*t*_(59)_ = 4.55, *p* < 0.001] at 20 days. As in the cortex, a correlation was calculated to compare relation of PGC1α expression in the hippocampus with body weight (Figure 6F). A significant correlation was found for rathletes [*r* = 0.36, *p* = 0.02], but not for sedentary rats [*r* = −0.34, *p* = 0.08].

**Figure 7:**
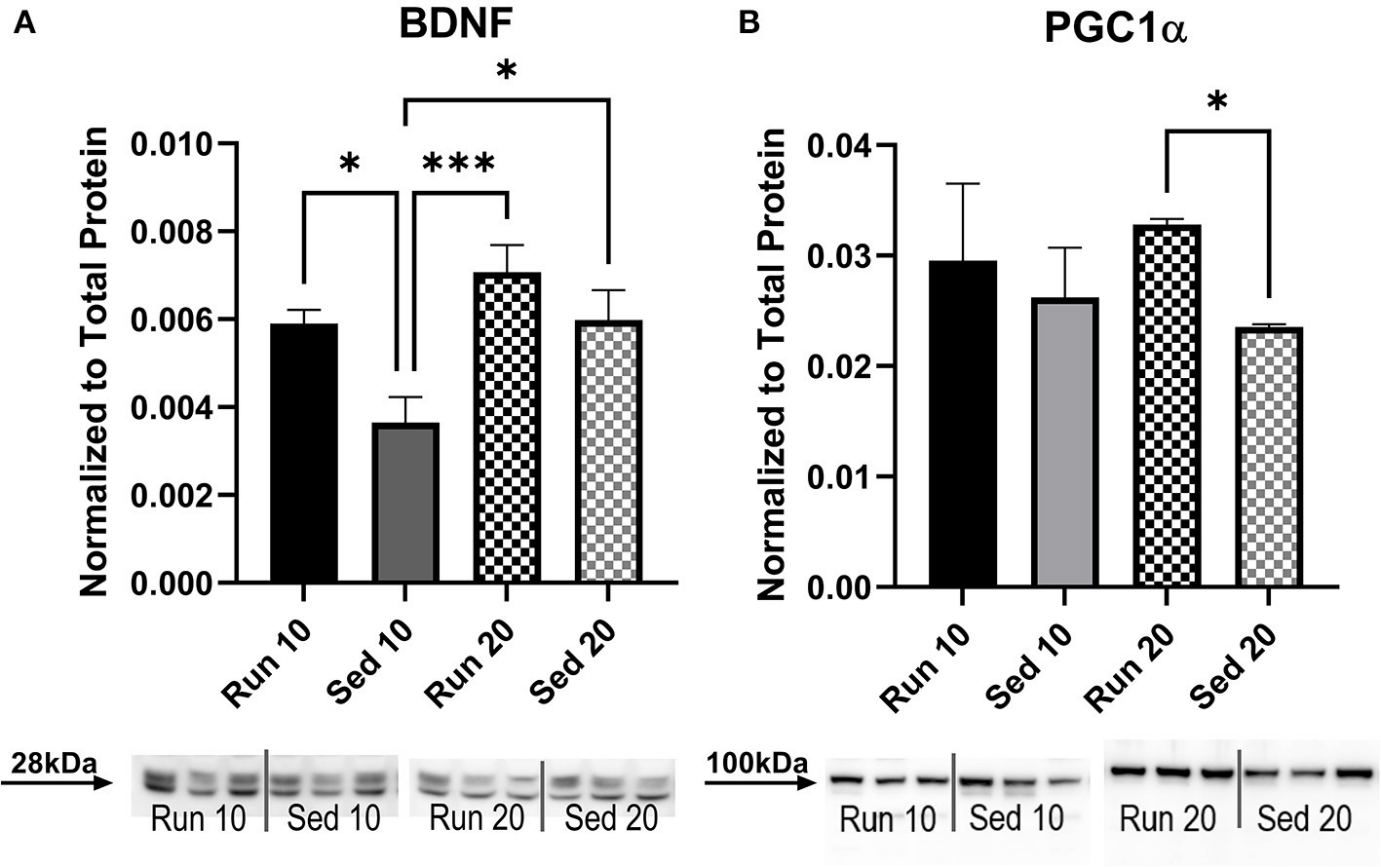
Muscle Protein Expression. A Western blot was used to determine levels of **(A)** BDNF and **(B)** PGC1α in the gastrocnemius muscle. Density of specific bands were normalized to total protein by Sypro Ruby staining (see Supplementary Figure 1). No sex differences were present so male and female data were combined. Acute (10 day) and chronic (20 day) changes in protein expression were compared between rathletes (“Run”) and sedentary (“Sed”) rats at these time points. Run 10 = voluntary running wheels for 10 days (*n* = 28), Sed 10 = locked wheels for 10 days (*n* = 28), Run 20 = voluntary running for 10 days then rested in homecage 10 days (*n* = 28), Sed 20 = locked wheels for 10 days then rested in homecage for 10 days (*n* = 28). Data are presented as average ± SEM. *p < 0.05, ***p < 0.001.

The gastrocnemius muscle was also analyzed for levels of BDNF (Figure 7A) and PGC1α (Figure 7B). A Two-way ANOVA for BDNF showed significant difference for activity [*F*_(3,78)_ = 5.56, *p* = 0.002], but not interaction [*F*_(3,78)_ = 0.91, *p* = 0.44]. Similar results were seen with PGC1α [Activity: *F*_(3,79)_ = 3.74, *p* = 0.14, Interaction: *F*_(3,79)_ = 1.34, *p* = 0.27]. There were no sex differences for either BDNF [*F*_(1,78)_ = 0.74, *p* = 0.39] or PGC1α [*F*_(1,79)_ = 3.92, *p* = 0.051] therefore, male and female groups were combined. Sedentary rats showed significantly less BDNF expression than rathletes at day 10 [*t*_(78)_ = 3.73, *p* = 0.049] and day 20 [*t*_(78)_ = 5.65, *p* < 0.001], and sedentary rats at day 20 [*t*_(78)_ = 3.86, *p* = 0.039]. There were no differences found at 10 days for PGC1α, but sedentary rats at 20 days showed significantly less expression than rathletes at 20 days [*t*_(79)_ = 3.15, *p* = 0.01]. Correlation coefficients were calculated for body weight and PGC1α expression, but were not significant for either rathletes [*r* = 0.09, *p* = 0.59] not sedentary groups [*r* = −0.03, *p* = 0.84].

### BDNF Bands

A Western blot technique was used to measure the multiple isoforms of BDNF with three different antibodies from Abcam (UK), Santa Cruz Biotechnology (CA, USA), and Novus Biologicals (CO, USA) (Supplementary Table 1). The Abcam antibody targeted the 38kDa band of BDNF most robustly, though many bands were observed (Supplementary Figure 2). Previous studies have focused analysis on the 14kDa dimer, however. The Santa Cruz antibody targeted multiple isoforms between 25–37kDa and no band at 14kDa was observed. The Novus antibody, primarily used in this study, targeted the 28kDa band most robustly, with a faint single band at the 14kDa band. The variability of band detection and the results comparing rathletes to sedentary males varied wildly between these antibodies emphasizing the importance of methodology in data analysis.

### Corticosterone

**Figure 8:**
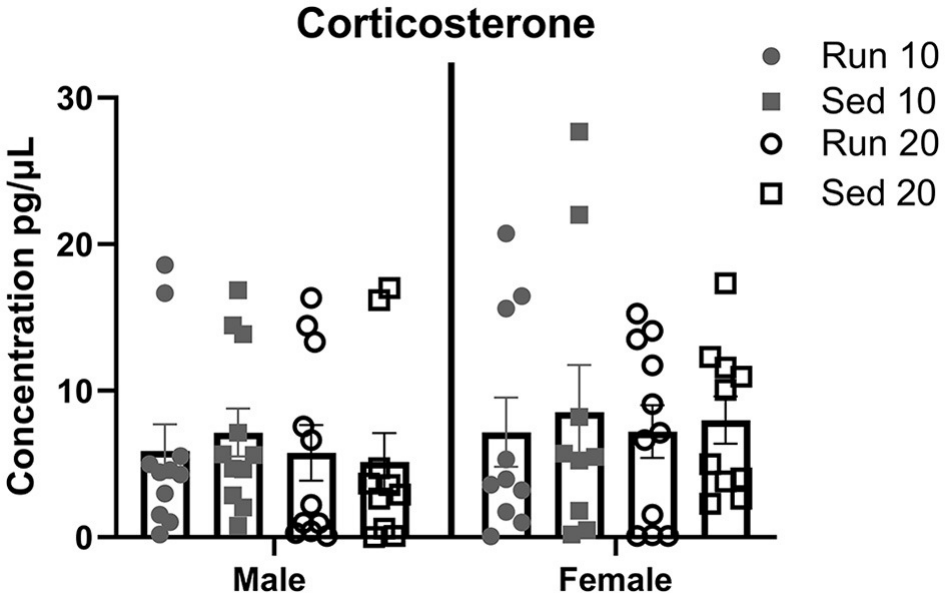
Plasma Corticosterone. Male and female rats were placed in a locked or functional running wheel for 10 days and then euthanized. A 2nd cohort of rats were placed in a functional or locked running wheel for 10 days then rested in their homecage for an additional 10 days before euthanasia. Truck blood was collected at time of euthanasia and plasma was isolated. Corticosterone level were determined by ELISA. Run 10 = voluntary running wheels for 10 days (*n* = 14/sex), Sed 10 = locked wheels for 10 days (*n* = 14/sex), Run 20 = voluntary running for 10 days then rested in homecage 10 days (*n* = 14/sex), Sed 20 = locked wheels for 10 days then rested in homecage for 10 days (*n* = 14/sex). Data are presented as average ± SEM.

Plasma corticosterone levels were measured from trunk blood at 10 and 20 days in males and females to determine effect of exercise as a stressor (Figure 8). A total of four samples were removed from analysis due to hemolysis (1 male sedentary 20 day, 1 female rathlete 10 day, 1 female sedentary 10 day, 1 female sedentary 20 day). A Two-way ANOVA found no significant differences for sex [*F*_(1,75)_ = 1.45, *p* = 0.23], activity [*F*_(3,75)_ = 0.20, *p* = 0.90], nor an interaction [*F*_(3,75)_ = 0.06, *p* = 0.98].

## Discussion

The results of this study have shown behavioral and physiological differences between active and sedentary adolescent rats. Voluntary running distance, duration, and intensity increased daily in males and females, though body weight gain did not differ between rathlete and sedentary groups. Sex differences in these groups were present. Distance and duration of running increased to a greater extent in female adolescent rats. This did not seem to be impacted by changes in female sex hormones across the estrous cycle as running behavior was not correlated to stage of estrous cycle. Cycling did not begin on day 1 of the study in 28% of females (23% of rathletes, 33% sedentary). Both sedentary and exercised female rats presented irregular cycling that was not significant across groups. This study also found sex and activity-dependent differences in protein expression of BDNF and PGC1α. There were exercise-dependent increases in BDNF in muscle tissue, and time-based changes in PGC1α. Finally, no sex or activity-dependent changes in basal corticosterone levels were observed. These results are important to further our understanding on the effects of exercise on the developing adolescent brain.

One significant strength of this study is that data were collected from two universities. Given the differences between the sites, increased variability was expected in the data (23, 24). The results, then, have a greater propensity for reproducibility as the data was representative of the variability seen between most laboratories. Further, this has translational potential. Clinically, variability exists in patient population, equipment, and tests performed in clinics and hospitals around the world. The current study intended to represent the clinical variability through the differences present at Pepperdine and UCLA. Another strength of the study is that the sedentary rats in were not completely inactive, often climbing in their wheels. As placement in running wheel cages occurred only 8 h per day, all animals had access to socialization and play when not in cages with locked or functional wheels. This is important as it relates clinically. Adolescents that are considered sedentary are not completely immobile, but rather spend little time (<3 h/week) exercising (26).

Activity-dependent differences in protein expression were observed with greater muscle expression of PGC1α in both males and females compared to sedentary groups. This could suggest differences in body composition, though not studied here. Studies in mice have found greater fat oxidation and mitochondrial biogenesis when an overexpression of PGC1α was observed (18, 27). Increased expression of skeletal muscle PGC1α, similar to results observed here, is important to fuel muscle fibers and promote endurance during exercise. Sex may be an important factor that influences exercise-induced changes in PGC1α, though pre-clinical research on adolescents that focus on this is limited. Due to the voluntary running paradigm implemented in the current study, increases in mitochondrial biogenesis and PGC1α in exercised animals were expected. This study found chronic protein expression increase in both males and females, with no sex differences. Acute exercise-dependent changes in PGC1α were not seen. Both sedentary and exercised rats showed increased protein levels in the parietal cortex and hippocampus at the chronic (20 day) time point compared to sedentary and exercised rats at the acute (10 day) time point. This suggests that the increase in PGC1α is due to development, possibly related to increases in body weight and size. For rathletes, the expression of PGC1α in each the cortex and hippocampus was positively correlated with body weight and to each other. A positive correlation was only observed between body weight and PGC1α in the cortex for the sedentary group. This suggests that exercise during development can enhance changes in PGC1α. Future studies should look at later time points to see if hippocampal expression of PGC1α ever correlates with body weight and/or levels in other brain regions in sedentary rats. It would be interesting to determine if the correlations with the hippocampus are specifically due to exercise and rathletes in this study and if it relates to exercise-induced neuroplasticity and neuroprotection following a brain insult. Previously, we have shown a significant increase in PGC1α in the hippocampus and parietal cortex in adolescent male rathletes after 20 days of voluntary wheel running with protection against cognitive deficits following brain injury (28). Similarly, another study allowing juvenile rats 20 days of voluntary exercise found that gene expression of PGC1α was only increased in the hippocampus of exercised females, and was actually decreased in the prefrontal cortex of males (29). However, prior research on 8 week old male mice found increases in PGC1α in various brain regions following 8 weeks of forced treadmill activity (18). Differences between these studies may be due to voluntary vs. forced exercise, intensity run, or length of exercise regimens.

This is the first study to look at sex as an influence on running intensity and duration. Other studies have found changes in activity that were dependent on sex hormones in both male and female rats. Specifically, castrated adult male rats or ovariectimized adult female mice given testosterone or 17β estradiol, respectively, increased running wheel activity (30–32). Another study looking at regularly cycling adult female rats found maximal running wheel activity during estrus (33). The current study did not find any correlation in running distance and estrous cycling in adolescent females. The role of testosterone in running behavior was also not studied. The differences in results may be in part due to age of the rats studied. Previous studies of estrous cycling and running have been performed on adults, not adolescents. Additionally, previous studies allowed voluntary exercise 24 h/day, while in the current study, time in wheels was limited to 8 h/day to allow for socialization with peers. It's important to note as well that 23% (5/22) of the female rats in the current study given access to a running wheel were not cycling on day 1 of wheel access. An additional six rats had at least one irregular cycle during access to running wheel. As hormone regularity in female adolescents, and its relation to running, is largely unstudied, it is uncertain whether these results are typical. It may be that these results are specific to adolescents as the hypothalamo-pituitary-gonadal (HPG) axis is starting to become active during puberty.

The key element in this study is that rats were given voluntary access to a running wheel. Rats were able to control running intensity and duration, which may influence physiological and neural changes. The average intensity of running for males was 7–21 m/min, and 9–23 m/min for females. Each bout of running was 5 min on average for males and 6–9 min for females. No sex differences were observed for running intensity. Intensity of exercise affects the energy stores being utilized by the body and the physiological changes that can occur as a result. For spurts of energy <1 min, anaerobic energy metabolism is favored (2). In this study, wheel revolutions run were counted every 1 min of time, the cut off for anaerobic exercise, so for all running analyzed in this study at least 60% of the energy system was using oxidation (3, 34). The importance of this is the corresponding response of the cardiovascular and respiratory system which can protect individuals from cardiac disease and stroke (35). This is important in terms of brain injury, where we have previously shown a reduction in PGC1α in adolescents following repeat TBI suggesting metabolic dysfunction that can be recovered with exercise. Also, in comparison to forced exercise, voluntarily running rats have been shown to have lower corticosterone levels, run at higher intensities, and have better memory performance on a spatial memory task (36, 37). In the current study, corticosterone levels ranged between 20 and 282pg/μl and variability was similar across groups. The range observed could be due to handling procedures in the study, circadian fluctuations, and timing of euthanasia. The levels observed in this study are consistent with non-stressed adolescent rats found by others (38, 39).

One unexpected result of this study was that levels of BDNF in the parietal cortex and the hippocampus were similar for running and sedentary rats and may be due to age of the rats or length of exercise regimen. Exercise has been shown to serve as a neuroprotectant for brain injury, preventing memory dysfunction, through the upregulation of BDNF (28). Previous studies of juvenile male and female rats found a significant increase in gene expression of BDNF in the hippocampus but not parietal cortex following 20 days of voluntary wheel running (29). Timing of the previous study may be a factor as the rats were analyzed closer to adulthood at 58 days of age compared to 45 days here. This study focused on adolescents whose brain regions are still maturing. During this time, BDNF has shown regional differences in expression beginning at low levels and increasing as regions mature (40). Specifically, in the hippocampus, BDNF mRNA levels in rats were low at 1 week and peaked at 4-5 weeks of age and then dropped again in adulthood (41). In the cortex, however, increases in BDNF were seen at 2 weeks and remained stable until adulthood (41). In the current study, BDNF was analyzed at 4–5 weeks of age, when BDNF mRNA in the targeted regions were already at their highest expression. This may explain the insignificant increases observed due to exercise.

One strength of this study was that intensity of running was monitored, and is described as the most important factor in aerobic fitness. Moderate intensity exercise is typically prescribed to promote cognitive health (particularly memory), cardiovascular health, and prevent metabolic diseases (35, 37). Running intensity has also been shown to influence BDNF expression. One week of low intensity forced running on a treadmill in adolescent rats resulted in greater BDNF mRNA expression than moderate-high intensity running (42). Low intensity running was described as 5 m/min for the first 5 min up to 11 m/min, while moderate-high intensity was defined by 8 m/min for 5 min up to 22 m/min. As intensity of running increased, hippocampal BDNF mRNA decreased with only those running at low intensity showing levels significantly greater than control rats (42). Average intensity running for the male and female rathletes in this study was high, ranging from 10–20 m/min (range 4.14–40.45 m/min). The inverse relationship between running intensity and BDNF transcription could explain the lack of significance in hippocampal BDNF protein expression found in this study. This is important in understanding how exercise can influence and protect the brain, particularly during adolescence.

The method to study changes in BDNF is important as well. Changes in gene expression do not directly correlate to changes in protein expression. In a clinical study of 12 male participants (age 23–38), Siefert et al. compared a 3 month exercise routine to a sedentary one (19). Plasma BDNF did not change in response to endurance training even though mRNA levels increased in the brain (19). They also found that acute (30 min, ~65% VO_2max_) exercise did not change plasma levels of BDNF from pre-exercise levels. In their study, mRNA of BDNF was studied and showed an increase, though protein expression was not measured. In a similar study of mice, BDNF mRNA was increased as well, but no increase was seen in protein levels, highlighting that gene transcription does not directly relate to protein translation (43). The current study measured protein expression of BDNF following 10 days of voluntary exercise. It may be that the results found were due to a combination of age, running patterns, and method to analyze BDNF. Future studies should consider such details to understand the physiological effects of exercise on BDNF in the brain.

One last possible explanation for the stable protein expression of BDNF is the stable expression of PGC1α in the brain and muscle. PGC1α has been shown to facilitate the release of BDNF through activation of FNDC5, a positive regulator of BDNF (17). Both PGC1α and FNDC5 are highly expressed in the brain and skeletal muscle, though little is known on the function of FNDC5 in the brain. Following 30 days of voluntary running wheel access, muscle and hippocampal expression of PGC1α and FNDC5 were upregulated, but was stable in the rest of the brain (17). Further, in PGC1α knockout mice, BDNF gene expression was significantly reduced in the cortex and hippocampus indicating that PGC1α is necessary for BDNF expression (17). Given that the current study did not find exercise-induced increase of PGC1α in the brain, and PGC1α is required for BDNF expression, then it is not unexpected to see the stable BDNF protein levels in the brain. The cognitive consequences of the stability warrant further investigation to understand how the employed exercise regimen could serve as a neuroprotectant.

Another important aspect of this study is the careful monitoring of the estrous cycle. The proportion of irregular menstrual cycles in female rats observed in this study was 13% at UCLA and 33% at Pepperdine. Clinically, one study of adolescents from Singapore found an average of 23.1% females with irregular menstrual cycles lasting either <22 days or more than 35 days (44), and another from India found rates at 24% (45), though other clinical studies have reported much higher percentages of irregularly cycling female adolescents (46–48). Irregular cycling may be due to an immature HPG axis (48, 49). Exercise can modulate the HPG axis through suppression of gonadotropin release (50). Clinical studies examining irregular menstrual cycling and distance running have found conflicting results, some finding dysfunction (anovulation) associated with increased mileage run (average age: 29), while others have not (49). Some studies of female runners, particularly focused on distance running, have been correlated with hormone imbalance and changes in menarche (11, 51). Lower estradiol and progesterone levels and shorter luteal length has been found in runners (average age: 23) with both regular and irregular cycles (49, 52). Research on young adult runners (average age: 30) have shown reduced progesterone levels even in the absence of irregular menstruation (51). Still others have found no difference in sex hormone levels of eumenorrheic and amenorrhoeic runners (53). In addition to a dysfunctional HPG axis, studies have found significantly lower systolic blood pressure in irregular cycling adolescent girls who participated in regular exercise (48). Following a mild brain injury, pituitary and hormone abnormalities are common, with 4–71% of males and 25–50% of females reporting irregularity in sexual function (54, 55). Females with a mild injury are 70% more likely to have sexual dysfunction compared to those severely injured as well (56). Knowing a baseline incidence of sexual dysfunction may help to better predict occurrence of sexual or hormonal abnormalities following brain injury and other brain disorders.

The large proportion of irregularly cycling rats is an important aspect to understand and consider for future studies. This is particularly important in studies relating to TBI. Previously we have shown that TBI causes a disruption in pituitary and endocrine function in adolescents (57). Pubertal hormones influence bone growth and thus can influence physical injuries during adolescence (2, 58, 59) with a transient decrease in bone strength correlating with peak height velocity, as evidenced by peak radial bone fractures in 12 yr old girls and 14 yr old boys (60). Irregularity in menstrual cycling has been found to influence adrenal functioning and stress responses in women, with increased serum cortisol in athletic-dependent menstrual dysfunction (11, 61–63). Sex hormone levels were not monitored in this study, but may relate to the high variability in corticosterone in female rathletes and should be considered in future studies. In animals already having hormonal dysfunction, responses to TBI could be exaggerated.

Irregular cycling may also may be due to inadequate energy intake relative to exercise energy expenditure, a deficiency now termed Relative Energy Deficiency in Sport (RED-S). Low energy intake has also been linked to metabolic rate, protein synthesis, growth, development, and other physiological processes (15, 16, 64). This impairment can affect males and well as females, and both non-athletes and athletes. When energy expenditure is greater than energy availability, the body will suppress functions not critical for survival like metabolic rate and reproduction, resulting in sex hormone dysfunction (15, 16, 65). In rodents, this idea is difficult to study. It would require monitoring daily food intake by individual rats, which would then require isolated housing, a stress for social animals, and adolescent rats in particular. Other, indirect measurements to suggest RED-S in an animal cohort could include tracking weight gain, heart rate, bone density, and neuroendocrine alterations including leptin, ghrelin, insulin-like growth factor-1, insulin, and sex hormones, which can be adversely affected by low energy availability (15, 66–69). It is an important consideration in overall adolescent health, and may make them susceptible to peripheral injuries and cardiovascular disease, which could also lead to stroke.

Studies focusing on irregular hormone cycling in males, particularly adolescent athletes, are severely lacking. Previous studies in males (average age: 23) have shown peaks of testosterone (T, 6.4 nmal/dL) every 112 min, follicular stimulating hormone (FSH, 0.38 IU/L) every 85 min and luteinizing hormone (LH, 1.3 IU/L) every 95 min and peak times correlate so that changes in testosterone levels can be predicted through concentration of LH 10–20 min earlier (70). Reports on the relationship between LH and T in male athletes are conflicting. In a study of young adult males, serum LH increased significantly as strength endurance exercise increased, though serum T remained steady (71). However, when endurance training weaned, serum T decreased and cortisol increased significantly. Male endurance runners (average age: 34) with over 65 km/week have shown lower serum testosterone levels (11, 72) and may be due to higher cortisol levels (73). While another study comparing male marathon runners to lean healthy controls (aerobic exercise 1 x/week) found increased LH in marathon runners but no difference in FST or T (74). Another study looking at male athletes found higher systolic and diastolic blood pressure in high intensity athletes (8 h/week, 5 h/week), along with lower LH levels and higher plasma T and estradiol compared to low intensity (30 min/week) males (75). While studies investigating effects of intensity are lacking, they may influence prevalence of hormonal dysfunction and metabolic disorders.

### Limitations

It's important to note that the analysis of BDNF targets may influence results. As previous studies have shown, different changes are seen in BDNF mRNA when compared to protein expression (43). Further, there are multiple cleavage products of BDNF, (a) a pro-BDNF precursor metabolite of 34kDa that gets processed into (b) mature BDNF of 14kDa, and (c) unprocessed BDNF at 28kDa (76) that can be targeted by ELISA and Western blot. Each of these metabolites has slightly different roles (77). The 34kDA pro-BDNF precursor is thought to be involved in long-term depression and apoptosis, while the 14kDa mature BDNF promotes long-term potentiation and cell survival, and the 28kDa is poorly understood but is thought to be released in an activity-dependent manner and signal through different receptors than the mature BDNF (76–78). With this in mind, the data analyzed play a strong role in the takeaway results, particularly as they relate to cognitive and behavioral changes. The antibody used in this study was specific to the 28kDA BDNF protein, which was acknowledged as mature BDNF. It may be beneficial to include analyses that target multiple variants of BDNF to better understand the changes that occur through exercise and other manipulations.

## Conclusions

Adolescence represents a unique period of development characterized by changes in body size, sex hormones, and physiology. Activity during this time can influence neurophysiology and behavior. This study has shown that 10 days of voluntary running leads to acute increased protein expression of BDNF in skeletal muscle and chronic increase in PGC1α. Voluntary running does not raise corticosterone levels and does not seem to be a stressor when adolescent rats are allowed socialization with peers. Sex differences were found whereby females ran further distances and for longer durations than males. Females also showed greater BDNF expression in the parietal cortex at the acute time point compared to the chronic time point, when rats were allowed rest for 10 days. This has implications for other studies interested in exercise or BDNF-dependent changes, and emphasizes the need to include sex as a dependent factor in analysis. Further, the adolescent period responds differently to exercise than adults. Behavioral outcomes may need to be adjusted for the adolescent age range given non-significant changes from exercise on neurophysiology. Data in this study were collected at two different sites to better represent rat populations across laboratories. The results presented here are thought to be robust and allow other studies to replicate the conclusions.

## Data Availability Statement

The raw data supporting the conclusions of this article will be made available by the authors, without undue reservation.

## Ethics Statement

The animal study was reviewed and approved by UCLA Animal Research Committee.

## Author Contributions

TG, MF, CG, and MP conceived the ideas presented and developed the behavioral paradigms used in this study. LF, HR, and RS performed the experiments and analyses. LF wrote the initial draft of the manuscript. All authors contributed to the article and approved the submitted version.

## Conflict of Interest

The authors declare that the research was conducted in the absence of any commercial or financial relationships that could be construed as a potential conflict of interest.
