# Supplementary material for: Sex Differences in Neurophysiological Changes Following Voluntary Exercise in Adolescent Rats
Source: Front Neurol. 2021 Jul 22;12:685822. doi: 10.3389/fneur.2021.685822 (PMC8339288; doi:10.3389/fneur.2021.685822)
Supplement: Supplementary file 1 [file Table_1.DOCX]

Supplementary Material


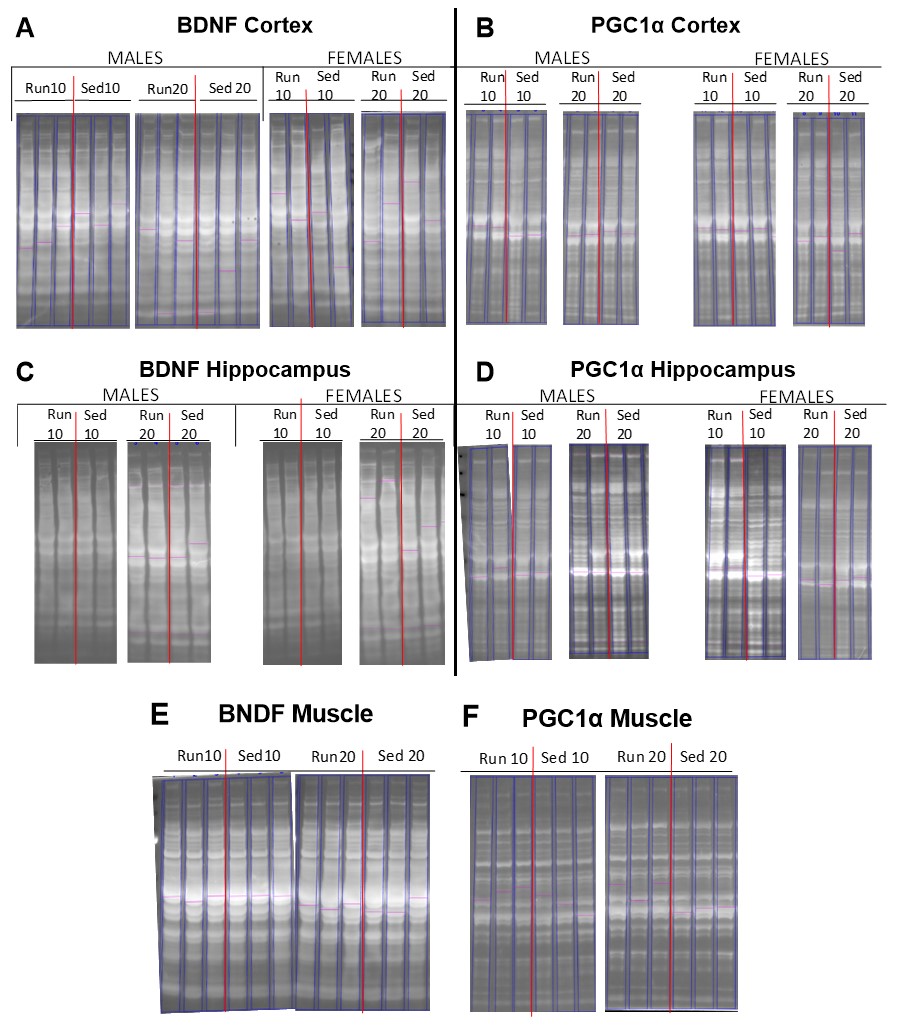
Supplemental Figure 1: Ruby Stain. Total protein loaded into lanes for Western blot were stained using Ruby, shown above, before antibodies were loaded to stain for specific proteins (See Figures 6 and 7). Western blots were run to analyze protein expression of BDNF and PGC1α and normalized to total protein in the parietal cortex (A, B), hippocampus (C, D), and muscle (E, F). Sex differences were not found in muscle, so data was combined. Ruby stains presented are relative to bands illustrated in Figures 6 and 7.


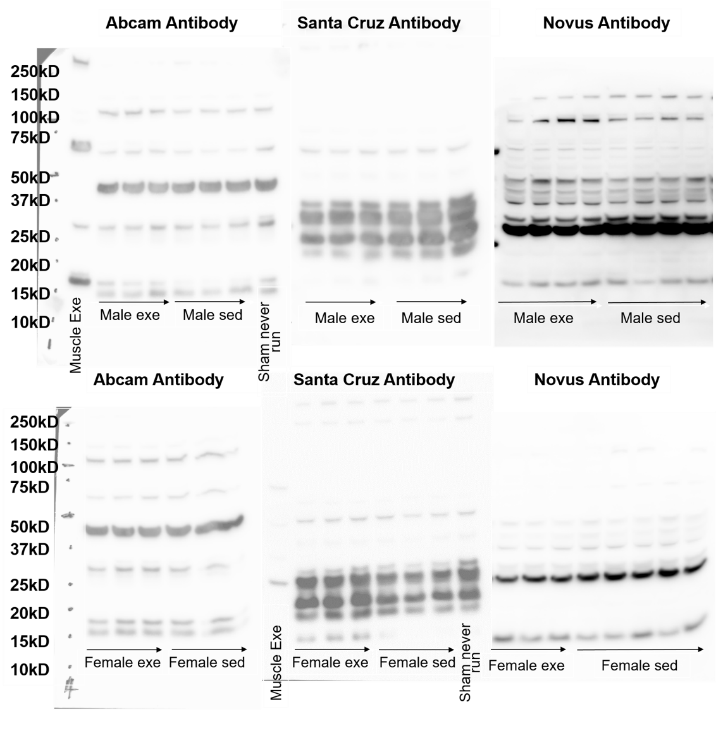


Supplementary Figure 2: Analysis of Various BDNF Antibodies. The BDNF protein has multiple cleavage products that can be detected through Western blotting techniques. Antibodies to detect BDNF can target multiple or specific isoforms. We tested BDNF antibodies from three different companies: Abcam (UK), Santa Cruz Biotechnology (CA, USA), and Novus Biologicals (CO, USA). Samples from this study of male (top) and female (bottom) rats given 10 days in a running wheel (exe) or 10 days in locked wheel (sed) were analyzed for each antibody. We also included a sample from gastrocnemius muscle of an exercised male from this study, and male sham rat that was sedentary for 20 days (from previous study – to compare across studies).

Supplemental Table 1: Primary Antibody Description. BDNF is a protein with multiple isoforms. These antibodies were used to test the detection of multiple BDNF bands.*antibody has been discontinued and replaced with pro-BDNF antibody (5H8): sc-65514 for detection of BDNF preproprotein (mouse monoclonal).

| **Product Name and Code** | **Host** | **Dilution** | **Detection** | **Supplier** |
| --- | --- | --- | --- | --- |
| Recombinant anti-BDNF [EPR1291] ab108319 | Rabbit monoclonal | 1:5000 | Mature form at ~14kDa and dimer at ~28kDa | Abcam, UK |
| BDNF Antibody (N-20) sc-546* | Rabbit polyclonal | 1:5000 | Precursor and mature BDNF | Santa Cruz Biotechnology, CA, USA |
| BDNF Antibody NB100-98682 | Rabbit polyclonal | 1:2000 | Specific to mature BDNF ~28kDa | Novus Biologicals, CO, USA |
